# Characteristics and Comparison of Adverse Events of Coronavirus Disease 2019 Vaccines Reported to the United States Vaccine Adverse Event Reporting System Between 14 December 2020 and 8 October 2021

**DOI:** 10.3389/fmed.2022.826327

**Published:** 2022-04-05

**Authors:** Chenyu Zou, Xiangzhong Xue, Jingjing Qian

**Affiliations:** Department of Health Outcomes Research and Policy, Harrison School of Pharmacy, Auburn University, Auburn, AL, United States

**Keywords:** COVID-19 vaccine, VAERS, adverse event reporting, vaccine safety, surveillance

## Abstract

**Background:**

This study assessed and compared the frequency and type of adverse events (AEs) of the Pfizer-BioNTech, Moderna, and Janssen coronavirus disease 2019 (COVID-19) vaccines reported in the Vaccine Adverse Event Reporting System (VAERS).

**Methods:**

A retrospective analysis examined VAERS reports between 14 December 2020 and 8 October 2021 and focused on AE reports related to COVID-19 vaccines and AE outcomes [e.g., emergency room (ER) visits after being vaccinated, hospitalization, prolongation of existing hospitalization, life-threatening events, disability, birth defect, and death]. Reporting odds ratios (RORs) and Breslow-Day statistics were used to compare AE reporting between COVID-19 and non-COVID vaccines and between individual COVID-19 vaccines.

**Results:**

A total of 604,157 AEs of COVID-19 vaccines were reported, including 43.51% for the Pfizer-BioNTech vaccine, 47.13% for the Moderna vaccine, and 9.12% for the Janssen COVID-19 vaccine. About 12.56% of patients visited ER after being vaccinated, 5.96% reported hospitalization, and 1.52% reported life-threatening events. Among the number of death cases (*n* = 7,674; mean age = 73), 2,025 patients (26.39%) had hypertension and 1,237 (16.12%) patients had cancer. RORs between COVID-19 vaccines and non-COVID vaccines identified increased ROR in ER visits, hospitalization, and life-threatening events. The results of the Breslow-Day statistics indicated heterogeneities between the disproportionality of reports across the four serious AE outcomes (i.e., ER visits, hospitalization, life-threatening events, and disability) between individual COVID-19 vaccines.

**Conclusion:**

Most current VAERS reports showed that the most commonly reported AEs of COVID-19 vaccines were mild. Cases with a mortality outcome tended to occur in older adults with underneath conditions. Close ongoing surveillance in the safety of COVID-19 vaccines is critical and will inform the use of individual COVID-19 vaccines. Given the known limitations associated with the passive spontaneous reporting system, such as VAERS, our findings need to be further assessed and verified through longitudinal, large healthcare data systems.

## Introduction

As of 29 October 2021, three vaccines are authorized for use in the United States (US) against SARS-CoV-2, the virus causing coronavirus disease 2019 (COVID-19) (Supplementary Table 1). The US Food and Drug Administration (FDA) issued the Emergency Use Authorization (EUA) for the Pfizer-BioNTech COVID-19 vaccine on 11 December 2020 (1), for the Moderna COVID-19 vaccine on 18 December 2020 (2), and for the Janssen (Johnson and Johnson) COVID-19 vaccine on 27 February 2021 (3). The Pfizer-BioNTech and the Moderna COVID-19 vaccines are administered as 2-dose series [the Centers for Disease Control and Prevention (CDC) recommended some groups of people to get a booster shot after 6 months of the second dose] (1–3), while the Janssen COVID-19 vaccine requires only one dose (CDC recommended some groups of people to get a booster shot after 2 months of the first dose) (3). The Advisory Committee on Immunization Practices (ACIP) recommended the use of the Pfizer-BioNTech COVID-19 vaccine to prevent COVID-19 in individuals aged 5 and above (1, 3, 4), whereas the Moderna and Janssen COVID-19 vaccines were recommended among individuals aged 18 and above (2, 3, 5). As of 29 October 2021, a total of 419,020,753 doses of the COVID-19 vaccine had been administered in the United States, and 191,997,869 people were fully vaccinated (6).

Vaccines are one of the most affordable and widely used public health interventions (7, 8), and vaccination has been recommended as a critical protective behavior for COVID-19 (9–15). The most recent Kaiser Family Foundation (KFF) COVID-19 Vaccine Monitor showed that about 62% of United States adults had received at least one dose of a vaccine, but 13% of participants indicated that they definitely would not get a COVID vaccine (16). Vaccine hesitancy for COVID-19 has been reported due to concerns about safety, potential adverse events (AEs), and effectiveness of current vaccines (15, 17–20). To detect possible safety problems from the COVID-19 vaccines, the safety monitoring systems for these vaccines, which is “the most intense and comprehensive in United States history,” have been developed (21). It is run by the CDC, FDA, and other federal partners; this broad, continued, and extensive safety surveillance system uses several existing data sources and “additional layers” [such as V-safe, a smartphone-based “after vaccine health checker (22)”] for ongoing safety monitoring, including the Vaccine Adverse Event Reporting System (VAERS) (21, 23). VAERS is a passive surveillance system and relies on unsolicited AE reports from individuals (24). In contrast, active surveillance entails proactively collecting and rapidly analyzing reports pertaining to millions of records in large healthcare datasets (25). The US FDA is conducting active surveillance using the Sentinel Biologics Effectiveness and Safety (BEST) System and the Centers for Medicare and Medicaid Services (CMS) System, collaborating with other federal and non-federal partners (26). Although active surveillance systems usually generate higher quality data (i.e., high levels of completeness, validity, and timeline) compared with passive surveillance systems, passive surveillance systems, including VAERS, have provided timely evidence in supporting regulatory decision-making regarding COVID-19 vaccination in the United States (27–29). This study assessed the AE reporting of the three available COVID-19 vaccines in VAERS since the first administration on 14 December 2020 and compared the AE reporting among individual COVID-19 vaccines as of 8 October 8 2021.

## Materials and Methods

### Data Source

Vaccine Adverse Event Reporting System, co-managed by the CDC and FDA, is a nationwide early warning system aimed to detect potential safety issues for all vaccines approved in the United States (30). It is a spontaneous reporting system, and vaccine manufacturers, healthcare professionals, patients, and customers can submit an AE report to VAERS, regardless of the seriousness of the AEs or how likely the vaccine has caused the AEs (30–32). VAERS is not intended to determine whether a vaccination caused a health problem, but to detect unexpected and unusual AEs and address possible reporting clusters (33). This study was approved by the Auburn University Institutional Review Board (IRB).

### Coronavirus Disease 2019 Vaccine Adverse Event Reports

All reports of the three approved COVID-19 vaccines (i.e., Pfizer-BioNTech, Moderna, and Janssen) between 14 December 2020 and 8 October 2021 were identified through the public VAERS dataset (variable “VAX_TYPE”). In addition, the patient’s age, sex, types of AE (variable “VAERS SYMPTOM”), and seven AE outcomes, namely, emergency room (ER) visit, hospitalization, prolongation of existing hospitalization, life-threatening events, disability, birth defect, and death, were extracted. For each death case reported from the AE reports for the three COVID-19 vaccines, we also assessed the patient’s reported medical history and current conditions (variables “HISTORY” and “CUR_ILL,” respectively).

### Statistical Analyses

Descriptive analyses of frequencies and proportions of AE reports by patient’s age, sex, type of COVID-19 vaccine administered, and AE outcomes were conducted. We also summarized the top 10 types of reported AEs from COVID-19 vaccines by frequency, overall and by individual vaccines. To compare the safety and AE reporting of COVID-19 vaccines with all other vaccines, we calculated the reporting odds ratio (ROR) and 95% confidence intervals (95% CIs) for each assessed AE outcome during the same period between 14 December 2020 and 8 October 2021 for all three COVID-19 vaccines combined together and for each individual COVID-19 vaccine. Given the surge of AE reporting for COVID-19 vaccines into VAERS after the initial administration, a sensitivity analysis was conducted by calculating RORs for COVID-19 vaccines compared to all other vaccines during a wider time window between 1 January 2020 and 8 October 2021. The Breslow-Day statistics was conducted to assess the homogeneity of RORs of assessed AE outcomes between individual COVID-19 vaccines (34). A *p*-value < 0.05 from the Breslow–Day test indicated a significant difference in RORs for a specific event between two individual COVID-19 vaccines. All analyses were conducted using SAS version 9.4 (SAS Institute Inc., Cary, NC), and *p* < 0.05 was set as statistical significance.

## Results

Between 14 December 2020 and 8 October 2021, a total of 661,614 AE reports were submitted to VAERS. Among these reports, 604,157 (91.32%) reports were of COVID-19 vaccines. Among all COVID-19 vaccine-related reports, there were 262,883 (43.51%) reports pertaining to the Pfizer-BioNTech COVID-19 vaccine, 284,765 (47.13%) reports pertaining to the Moderna COVID-19 vaccination, 55,111 (9.12%) reports pertaining to the Janssen COVID-19 vaccine, and 1,308 (0.22%) reports with the missing manufacturer (Table 1). Among the reports of AEs due to COVID-19 vaccines, 412,610 (68.30%) were women, and the mean age was 49.43 years (in the range of 0.08–119 years). There were 75,911 (12.56%) patients who visited the ER after being vaccinated and 36,030 (5.96%) patients reported hospitalization with an average length of 3.11 days (in the range of 1–22 days). In addition, 9,193 (1.52%) individuals reported to be experiencing life-threatening events, 8,890 (1.47%) individuals reported disability, 7,674 (1.27%) individuals were reported as dead, 343 (0.06%) individuals reported birth defect, and 305 (0.05%) individuals had prolongation of existing hospitalization after being vaccinated (Table 1).

**TABLE 1:** Summary of COVID-19 vaccines adverse event (AE) reports to VAERS, 14 December 2020 to 8 October 2021.

VAERS AE reports	*N* (%)
**Number of AE reports of COVID-19 vaccines**	604,157
Pfizer-BioNTech COVID-19 vaccine	262,883 (43.51)
Moderna COVID-19 vaccine	284,765 (47.13)
Janssen COVID-19 vaccine	55,111 (19.73)
Reports with missing manufacturer	1,308 (0.22)
**Mean age (range), years**	49.43 (0.08–119)
0–18	26,454 (4.38)
19–44	200,776 (33.23)
45–64	186,073 (30.80)
65–84	119,189 (19.73)
85 +	12,776 (2.11)
Missing	58,889 (9.75)
**Female sex**	412,610 (68.30)
**AE outcomes**	
ER visits after being vaccinated	75,911 (12.56)
Hospitalization	36,030 (5.96)
Life-threatening events	9,193 (1.52)
Disability	8,890 (1.47)
Death	7,674 (1.27)
Birth defect	343 (0.06)
Prolongation of existing hospitalization	305 (0.05)

The top ten AE reports for all COVID-19 vaccines and individual COVID-19 vaccines by frequency are listed in Table 2. Headache, fatigue, chills, pyrexia, and pain were among the top five commonly reported AEs for all three COVID-19 vaccines, overall and individually.

**TABLE 2:** Top 10 adverse events of COVID-19 vaccines reported to VAERS by frequency, 14 December 2020 to 8 October 2021.

Top 10 adverse events of all three COVID-19 vaccines	*N* (%)
Headache	100,458 (16.63)
Fatigue	85,313 (14.12)
Chills	80,334 (13.30)
Pyrexia	74,047 (12.26)
Pain	64,924 (10.75)
Dizziness	61,925 (10.25)
Nausea	52,483 (8.69)
Pain in extremity	50,179 (8.31)
Arthralgia	36,192 (5.99)
Injection site pain	32,400 (5.36)
**Top 10 adverse events of Pfizer-BioNTech COVID-19 vaccine *N* (%)**	
Headache	41,122 (15.64)
Fatigue	35,407 (13.47)
Dizziness	29,812 (11.34)
Chills	29,620 (11.27)
Pyrexia	27,463 (10.45)
Pain	26,223 (9.98)
Nausea	22,474 (8.55)
Pain in extremity	19,040 (7.24)
Arthralgia	15,775 (6.00)
Dyspnea	14,637 (5.57)
**Top 10 adverse events of Moderna COVID-19 vaccine *N* (%)**	
Headache	46,037 (16.17)
Fatigue	40,479 (14.21)
Chills	40,030 (14.06)
Pyrexia	36,636 (12.87)
Pain	30,353 (10.66)
Pain in extremity	26,241 (9.21)
Dizziness	24,603 (8.64)
Nausea	23,682 (8.32)
Injection site erythema	20,207 (7.10)
Injection site pain	19,911 (6.99)
**Top 10 adverse events of Janssen COVID-19 vaccine *N* (%)**	
Headache	13,048 (23.68)
Chills	10,479 (19.01)
Pyrexia	9,735 (17.66)
Fatigue	9,251 (16.79)
Pain	8,177 (14.84)
Dizziness	7,396 (13.42)
Nausea	6,210 (11.27)
Pain in extremity	4,801 (8.71)
Arthralgia	3,268 (5.93)
Myalgia	3,190 (5.79)

It was reported that 7,674 individuals died after they were administered the COVID-19 vaccines with a mean age of 72.75 (range: 0.42–106), accounting for 1.27% of all AE reports of COVID-19 vaccines (Table 3). Among these death cases, 3,500 (45.61%) received the Pfizer-BioNTech COVID-19 vaccine, 3,329 (43.38%) received the Moderna COVID-19 vaccine, 814 (10.61%) received the Janssen COVID-19 vaccine, and 31 (0.40%) deaths with unidentified vaccine manufacturer. A total of 2,025 (26.39%) death cases indicated previous diagnoses of hypertension, and 1,237 (16.12%) death cases had cancer. Other common comorbidities included diabetes mellitus (DM) (10.65%), heart disease (9.47%), chronic obstructive pulmonary disease (COPD) (5.75%), transient ischemic attack (TIA) (5.72%), and heart failure (5.20%).

**TABLE 3:** Summary of death cases of COVID-19 vaccines reported to VAERS, 14 December 2020 to 8 October 2021.

Mean age (range), years	72.75 (0.42–106)
	**Number (%)**
**Number of death reports of COVID-19 vaccines**	7,674
Number of death reports of Pfizer-BioNTech COVID-19 vaccine	3,500 (45.61)
Number of death reports of Moderna COVID-19 vaccine	3,329 (43.38)
Number of death reports of Janssen COVID-19 vaccine	814 (10.61)
Number of death reports with missing manufacturer	31 (0.40)
**Medical history and current conditions for all death cases**	
Hypertension	2,025 (26.39)
Cancer	1,237 (16.12)
DM (Diabetes Mellitus)	817 (10.65)
Heart disease	727 (9.47)
COPD (Chronic Obstructive Pulmonary Disease)	441 (5.75)
TIA (Transient Ischemic Attack)	439 (5.72)
Heart failure	399 (5.20)
Hyperlipidemia	348 (4.53)
Dementia	316 (4.12)
Atrial fibrillation	315 (4.10)
Coronary artery	296 (3.86)
CHF (Congestive Heart Failure)	287 (3.74)
Atherosclerosis	272 (3.54)
Thyroid disease	269 (3.51)
CKD (Chronic Kidney Disease)	265 (3.45)
Obesity	199 (2.59)
Kidney disease	197 (2.57)
GERD (Gastroesophageal reflux disease)	195 (2.54)
Depression	179 (2.33)
Alzheimer’s disease	137 (1.79)
Dysphagia	107 (1.39)
Pulmonary*	87 (1.13)
PVD (Peripheral Vascular Disease)	48 (0.63)
COVID-19	42 (0.55)
Hypercholesterolemia	37 (0.48)
ASCVD (Atherosclerotic cardiovascular disease)	33 (0.43)
Schizophrenia	27 (0.35)
AAA (Abdominal aortic aneurysm)	23 (0.30)
Hypokalemia	11 (0.14)

*Identified based on reported terms of “pneumonia” or “pulmonary” from variables “HISTORY” and “CUR_ILL”.

During 14 December 2020 and 8 October 2021 (Table 4 and Supplementary Figure 1), RORs of all seven serious AE outcomes between COVID-19 vaccines and non-COVID vaccines indicated a potentially higher ROR in ER visits (ROR = 1.08, 95% CI = 1.08–1.09), hospitalization, (ROR = 1.21, 95% CI = 1.14–1.28), prolongation of existing hospitalization (ROR = 1.41, 95% CI = 1.01–1.96), and life-threatening events (ROR = 1.20, 95% CI = 1.07–1.35). The increased ROR in ER visits, hospitalization, and life-threatening events for COVID-19 vaccines was consistent with results from sensitivity analysis, but not for prolongation of existing hospitalization (ROR = 0.56, 95% CI = 0.42–0.75 in sensitivity analysis) and death (ROR = 1.87, 95% CI = 1.69–2.07 in sensitivity analysis, Table 4 and Supplementary Figure 2).

**TABLE 4:** Main and sensitivity analyses of RORs for COVID-19 vaccines, overall and by individual COVID-19 vaccine.

Main analysis: December 14, 2020–October 8, 2021				Sensitivity analysis: January 1, 2020–October 8, 2021			
Compare all three COVID-19 vaccines to other non-COVID vaccines				Compare all three COVID-19 vaccines to other non-COVID vaccines			
AE Outcomes	ROR	95% CI		AE Outcomes	ROR	95% CI	
ER visits*	1.08	1.08	1.09	ER visits*	1.08	1.08	1.08
Hospitalization*	1.21	1.14	1.28	Hospitalization*	1.43	1.37	1.49
Prolongation of existing hospitalization*	1.41	1.01	1.96	Prolongation of existing hospitalization*	0.56	0.42	0.75
Life-threatening events*	1.20	1.07	1.35	Life-threatening events*	1.73	1.59	1.89
Disability*	0.43	0.40	0.47	Disability*	0.53	0.50	0.56
Birth defect*	0.60	0.39	0.92	Birth defect	0.87	0.62	1.21
Death	1.11	0.98	1.25	Death*	1.87	1.69	2.07

**Compare Pfizer-BioNTech COVID-19 vaccine to all other vaccines**				**Compare Pfizer-BioNTech COVID-19 vaccine to all other vaccines**			

ER visits*	1.51	1.49	1.54	ER visits*	1.56	1.54	1.58
Hospitalization*	1.35	1.32	1.37	Hospitalization*	1.38	1.35	1.41
Prolongation of existing hospitalization	1.02	0.82	1.26	Prolongation of existing hospitalization	1.00	0.81	1.23
Life-threatening events*	1.09	1.05	1.14	Life-threatening events*	1.31	1.25	1.36
Disability*	1.25	1.20	1.30	Disability*	1.18	1.13	1.22
Birth defect*	1.35	1.10	1.65	Birth defect*	1.36	1.11	1.66
Death*	1.09	1.05	1.14	Death*	1.17	1.12	1.22

**Compare Moderna COVID-19 vaccine to all other vaccines**				**Compare Moderna COVID-19 vaccine to all other vaccines**			

ER visits*	0.66	0.65	0.67	ER visits*	0.69	0.68	0.70
Hospitalization*	0.70	0.69	0.72	Hospitalization*	0.74	0.72	0.75
Prolongation of existing hospitalization*	0.71	0.57	0.89	Prolongation of existing hospitalization*	0.71	0.57	0.88
Life-threatening events*	0.71	0.68	0.74	Life-threatening events*	0.76	0.73	0.79
Disability*	0.64	0.61	0.66	Disability*	0.62	0.59	0.65
Birth defect*	0.66	0.53	0.81	Birth defect*	0.68	0.55	0.84
Death*	0.87	0.83	0.91	Death*	0.93	0.89	0.97

**Compare Janssen COVID-19 vaccine to all other vaccines**				**Compare Janssen COVID-19 vaccine to all other vaccines**			

ER visits*	1.18	1.15	1.21	ER visits*	1.22	1.19	1.25
Hospitalization*	1.23	1.19	1.27	Hospitalization*	1.25	1.21	1.30
Prolongation of existing hospitalization*	1.43	1.03	1.99	Prolongation of existing hospitalization*	1.41	1.01	1.96
Life-threatening events*	1.40	1.32	1.50	Life-threatening events*	1.45	1.37	1.55
Disability*	1.09	1.01	1.16	Disability	1.05	0.98	1.13
Birth defect	0.93	0.64	1.35	Birth defect	0.94	0.65	1.36
Death*	1.19	1.10	1.28	Death*	1.24	1.15	1.33

*ROR, reporting odds ratio; CI, confidence interval; AE, adverse event.*

*Statistically significant based on 95% CIs.

For the comparison between the Pfizer COVID-19 vaccine and all other vaccines, RORs for six serious AE outcomes (i.e., ER visits, hospitalization, life-threatening events, disability, birth defect, and death) indicated a higher ROR in these serious events for the Pfizer COVID-19 vaccine during 14 December 2020 and 8 October 2021. These RORs remained statistically significant in a sensitivity analysis. For the Moderna COVID-19 vaccine vs. all other vaccines, RORs for all seven assessed serious AE outcomes indicated a lower ROR (indicating no reporting risk) for the Moderna COVID-19 vaccine during 14 December 2020 and 8 October 2021, as well as in the sensitivity analysis. For the Janssen COVID-19 vaccine vs. all other vaccines, RORs for the five assessed serious AE outcomes (i.e., ER visits, hospitalization, prolongation of existing hospitalization, life-threatening events, and death) indicated a higher ROR in these serious events for the Janssen COVID-19 vaccine in both main and sensitivity analyses (Table 4 and Supplementary Figures 1, 2).

The results of the Breslow-Day statistics to compare RORs of seven serious AE outcomes for individual COVID-19 vaccines with all other vaccines are shown in Table 5. We found that the signals of disproportionate reporting for the four serious AE outcomes (i.e., ER visits, hospitalization, life-threatening events, and disability) with each individual COVID-19 vaccine were all different from another individual COVID-19 vaccine (*p* < 0.05).

**TABLE 5:** Breslow-day tests for paired comparisons between individual COVID-19 vaccines.

COVID-19 vaccine comparisons	Main analysis: December 14, 2020–October 8, 2021		Sensitivity analysis: January 1, 2020–October 8, 2021	
	Outcomes	*P*-value	Outcomes	*P*-value
Pfizer-BioNTech vs. Moderna	ER visits*	<0.0001	ER visits*	<0.0001
	Hospitalization*	<0.0001	Hospitalization*	<0.0001
	Prolongation of existing hospitalization*	0.0245	Prolongation of existing hospitalization*	0.0276
	Life-threatening events*	<0.0001	Life-threatening events*	<0.0001
	Disability*	<0.0001	Disability*	<0.0001
	Birth defect*	<0.0001	Birth defect*	<0.0001
	Death*	<0.0001	Death*	<0.0001
Pfizer-BioNTech vs. Janssen				
	ER visits*	< 0.0001	ER visits*	<0.0001
	Hospitalization*	<0.0001	Hospitalization*	<0.0001
	Prolongation of existing hospitalization	0.09	Prolongation of existing hospitalization	0.08
	Life-threatening events*	0.0045	Life-threatening events*	0.0012
	Disability*	<0.0001	Disability*	<0.0001
	Birth defect	0.08	Birth defect	0.09
	Death	0.06	Death	0.17
Janssen vs. Moderna				
	ER visits*	<0.0001	ER visits*	<0.0001
	Hospitalization*	<0.0001	Hospitalization*	<0.0001
	Prolongation of existing hospitalization*	0.0005	Prolongation of existing hospitalization*	0.0006
	Life-threatening events*	<0.0001	Life-threatening events*	<0.0001
	Disability*	<0.0001	Disability*	<0.0001
	Birth defect	0.11	Birth defect	0.14
	Death*	<0.0001	Death*	<0.0001

*Statistically significant based on 95% CIs.

## Discussion

This study described the most up-to-date frequencies and types of AE reporting for the three approved COVID-19 vaccines (i.e., Pfizer-BioNTech, Moderna, and Janssen) and compared AE reporting rates among individual vaccines using the VAERS database between 14 December 2020 and 8 October 2021. Overall, the majority (over 68%) of AE reports about COVID-19 vaccines came from female patients. The reported AE outcomes involved large proportions of ER visits and hospitalizations after vaccination. However, most commonly reported AEs of COVID-19 vaccines were mild, and cases with a mortality outcome mainly occurred among older adults with underneath chronic conditions, such as hypertension, cancer, and DM.

Our findings are consistent with recently published review studies. A rapid review evaluated the safety profile of COVID-19 vaccines and reported that among the most common local reactions, pain at the injection site was the most common, while fatigue and headache were the most common systemic reactions. A very low frequency of serious AEs was reported (<0.1%) (35). Hernández and colleagues also reported that the most common AEs reported after the administration of three COVID-19 vaccines administered in Europe (i.e., Pfizer, Moderna, and Astra-Zeneca) included the injection site reactions (e.g., sore arm and erythema) and non-specific systemic effects (e.g., myalgia, chills, fatigue, headache, and fever). Most of these AEs occurred soon after vaccination and resolved quickly (36). Our findings using VAERS, a passive surveillance system, are also consistent with some prospective, active surveillance studies. For example, in a large-scale SARS-CoV-2 surveillance program in Madurai, India, the authors found that increased risk of COVID-19 infection (3.6%) and death among positive cases (2.4%) were associated with older age, male sex, and comorbidities, such as cancer, DM, other endocrine disorders, hypertension, other chronic circulatory disorders, respiratory disorders, and chronic kidney disease (37).

In addition, largely consistent results from our main and sensitivity ROR analyses indicated minor or maybe ignorable reporting bias during the study period of 14 December 2020 and 8 October 2021. Over 90% of AE reports in VAERS during this time period were related to COVID-19 vaccines, and our analysis included more than 600,000 AE events across the country. Many studies have shown that routine immunization services faced severe challenges and the number of immunizations fell in the year 2020, especially for children (38–40), which may explain the small proportion of AE events related to non-COVID vaccination in VAERS during our study period. Assessing a longer time span of VAERS reports might reduce potential reporting bias (41). However, the COVID-19 vaccines have undergone the most intensive safety monitoring in United States history (21, 23). Compared to all previous vaccines, the prioritization of vaccines was unique (42–44), and healthcare providers are required to report serious AEs to VAERS after COVID-19 vaccination under EUA (45). These factors may have led to increased reporting of COVID-19 vaccines and the completeness of AE reports in VAERS. Nevertheless, the large amount of AE reports for COVID-19 vaccines in VAERS provides a great opportunity for practitioners, policymakers, and researchers to timely monitor the safety of COVID-19 vaccines.

Our findings provided up-to-date AE reporting evidence for the three currently marketed COVID-19 vaccines. Results from the Breslow-Day statistics demonstrated differences in ROR of four serious AE outcomes (i.e., ER visits, hospitalization, life-threatening events, and disability) between individual COVID-19 vaccines. Specifically, different from the Pfizer-BioNTech and Janssen COVID-19 vaccines, the Moderna COVID-19 vaccine showed no increased reporting risks for these AEs compared to all other vaccines. Although the nature of the spontaneous reporting VAERS data can only support AE signal detection instead of demonstrating the causal association between vaccine administration and reported AE outcomes (24, 32), US CDC and FDA have updated some new guidelines based on the growing number of COVID-19 vaccine administrated and AE reports. For example, according to the CDC’s Morbidity and Mortality Weekly Report (MMWR), reports of syncope were approximately 164 times more common after the Janssen COVID-19 vaccination (8.2 per 100,000) than after influenza vaccination (0.05 per 100,000) (46). On 23 April 2021, CDC and FDA ended the pause on the use of the Janssen COVID-19 vaccine but suggested that women younger than 50 years should be aware of the rare risk of blood clots with low platelets (47). On 16 December 2021, the CDC’s ACIP held an emergency meeting to review the updated data on thrombosis with thrombocytopenia syndrome and an updated benefit-risk assessment. The ACIP made a recommendation for preferential use of mRNA COVID-19 vaccines (e.g., Pfizer-BioNTech and Moderna) over the Janssen COVID-19 vaccine, including both primary and booster doses administered to prevent COVID-19, for all persons aged ≥18 years (27). In addition, FDA approved the Pfizer-BioNTech COVID-19 vaccine for the prevention of COVID-19 disease in individuals aged 16 and above on 23 August 2021, (48) and further authorized it for children aged 5–11 years under a EUA on 29 October 2021 (1). Therefore, in time, continuous surveillance in existing, new, and serious safety problems of COVID-19 vaccines is critical.

It is critical to monitor the safety of authorized COVID-19 vaccines through both passive and active safety surveillance systems, which can detect and refine safety findings in a relatively rapid manner. Active surveillance systems provide the most accurate and timely information. For example, Australia’s AusVaxSafety system has been used to evaluate the safety profile of live-attenuated herpes zoster vaccine among older (70–79 years of age) Australian adults in the first two program years at 246 sentinel surveillance immunization sites, and the authors found that the rates of medical attendance were low (0.3%) with no safety signals identified (49). Our results highlight the importance of constant vigilance in order to quickly evaluate the safety of COVID-19 vaccines, given that we found higher ROR in ER visits and hospitalization for COVID-19 vaccines compared to other vaccines that have been routinely used for years. A pharmacovigilance analysis using the World Health Organization (WHO) international database (VigiBase) compared AE reporting with mRNA COVID-19 vaccines (Pfizer-BioNTech and Moderna) and influenza vaccines from 1 January 2020 to 17 January 2021 (50). The VigiBase study did not identify significant safety concerns regarding mRNA vaccination in real-world settings. The authors reported an overall lower risk of serious AEs following mRNA vaccines compared to influenza vaccines, and 103 (0.5%) deaths out of 18,755 COVID-19 vaccine-related AEs [compared to 104 (0.4%) deaths out of 27,895 influenza vaccine-related AEs] (50). Another study analyzed data from two passive surveillance systems, VAERS and European EudraVigilance, to compare the reporting rates of anaphylaxis, a severe, potentially life-threatening allergic reaction, between COVID-19 vaccines and other vaccines (51). The authors found that COVID-19 vaccines ranked fifth in reported anaphylaxis rates, behind rabies, tick-borne encephalitis, measles-mumps-rubella-varicella, and human papillomavirus vaccines (70.77, 20, 19.8, and 13.65 cases per 1,000,000 vaccine doses, respectively). Our analysis using VAERS included a larger number (more than 600,000) of AE reports related to COVID-19 vaccines compared to the VigiBase analysis. Our findings provide critical evidence to bring potential safety signals to the attention of public health professionals and policymakers.

Similar to most research using VAERS, our study also has some unavoidable limitations. First, VAERS is a passive spontaneous reporting system with substantial incomplete, inaccurate, and missing information, which could lead to under- or over-reporting, reporting biases, inconsistency in the quality of reports, and lack of denominator data and unbiased comparison groups (24, 30–33). For example, some mass vaccination sites reported more information than others (46). Second, the AE reporting date might not be the actual vaccination date and the duration of vaccination exposure varied in each AE report. In addition, we could not calculate the time between death and vaccination date due to a considerable amount of missing data on the date of vaccination or date of death in VAERS. Finally, the nature of VAERS data does not infer causality. VAERS accepts all reports without judging whether the event was caused by the vaccine (24, 30, 32, 33, 52). Our findings only identified signals or potential risks of AEs for signal generation and surveillance purposes. Future research using active surveillance systems including longitudinal, large healthcare data systems to verify safety signals identified through passive surveillance is warranted.

In conclusion, our findings provided up-to-date AE reporting evidence for the three United States marketed COVID-19 vaccines during the first 10 months of utilization. The most commonly reported AEs of COVID-19 vaccines were mild. Cases with a mortality outcome mainly occurred among older adults with underneath chronic conditions. We found differences in reporting of serious AE outcomes between individual COVID-19 vaccines. The preliminary evidence generated from this study needs to be verified through active surveillance systems in order to inform the selection of individual COVID-19 vaccines and guide current and upcoming COVID-19 vaccination in the United States and around the world.

## Data Availability Statement

Publicly available VAERS datasets were analyzed in this study. The data can be found here: https://vaers.hhs.gov/data/datasets.html?

## Ethics Statement

The studies involving human participants were reviewed and approved by the Auburn University Institutional Review Boards. Written informed consent for participation was not required for this study in accordance with the national legislation and the institutional requirements.

## Author Contributions

CZ: conceptualization, methodology, software, validation, formal analysis, data curation, writing (original draft), and visualization. XX: conceptualization, methodology, software, validation, formal analysis, data curation, writing (review and editing), and visualization. JQ: conceptualization, methodology, data curation, writing (original draft), and supervision. All authors contributed to the article and approved the submitted version.

## Conflict of Interest

The authors declare that the research was conducted in the absence of any commercial or financial relationships that could be construed as a potential conflict of interest.

## Publisher’s Note

All claims expressed in this article are solely those of the authors and do not necessarily represent those of their affiliated organizations, or those of the publisher, the editors and the reviewers. Any product that may be evaluated in this article, or claim that may be made by its manufacturer, is not guaranteed or endorsed by the publisher.
