# Supplementary material for: Characteristics and Comparison of Adverse Events of Coronavirus Disease 2019 Vaccines Reported to the United States Vaccine Adverse Event Reporting System Between 14 December 2020 and 8 October 2021
Source: Front Med (Lausanne). 2022 Apr 5;9:826327. doi: 10.3389/fmed.2022.826327 (PMC9016134; doi:10.3389/fmed.2022.826327)

Supplementary Materials

Supplementary Table 1. Approved COVID-19 Vaccines in the United States (As of October 29, 2021)

**COVID-19 Vaccines**	**Pfizer-BioNTech**	**Moderna**	**Janssen/J&J**
**Date of EUA**	December 11, 2020	December 18, 2020	February 27, 2021
**Recommended age range**	≥5	≥18	≥18
**Dose series**	2 doses*, 3 weeks apart	2 doses*, 4 weeks apart	1 dose**
**Total Administrated dose**	245,831,871	157,205,236	15,549,336
**Number of people fully vaccinated**	106,315,307	70,291,347	15,273,490

* Centers for Disease Control and Prevention (CDC) recommended some groups of people to get a booster shot after 6 months of the second dose.

** Centers for Disease Control and Prevention (CDC) recommended some groups of people to get a booster shot after 2 months of the first dose.

Supplementary Figure 1. Forest Plot of RORs for COVID-19 vaccines, overall and by individual COVID-19 vaccines (Main Analysis: December 14, 2020 - October 8, 2021)

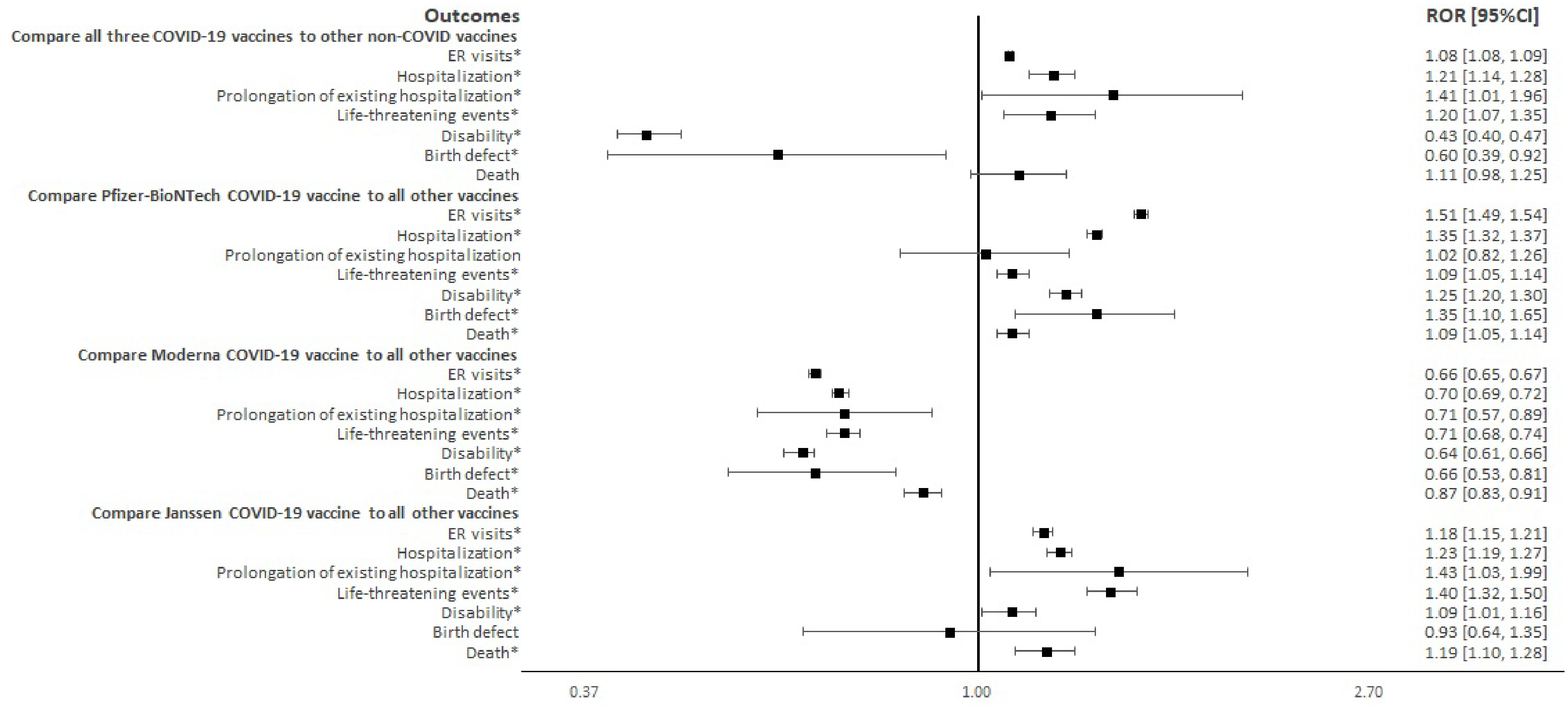

Supplementary Figure 2. Forest Plot of RORs for COVID-19 vaccines, overall and by individual COVID-19 vaccines (Sensitivity Analysis: January 1, 2020 - October 8, 2021)

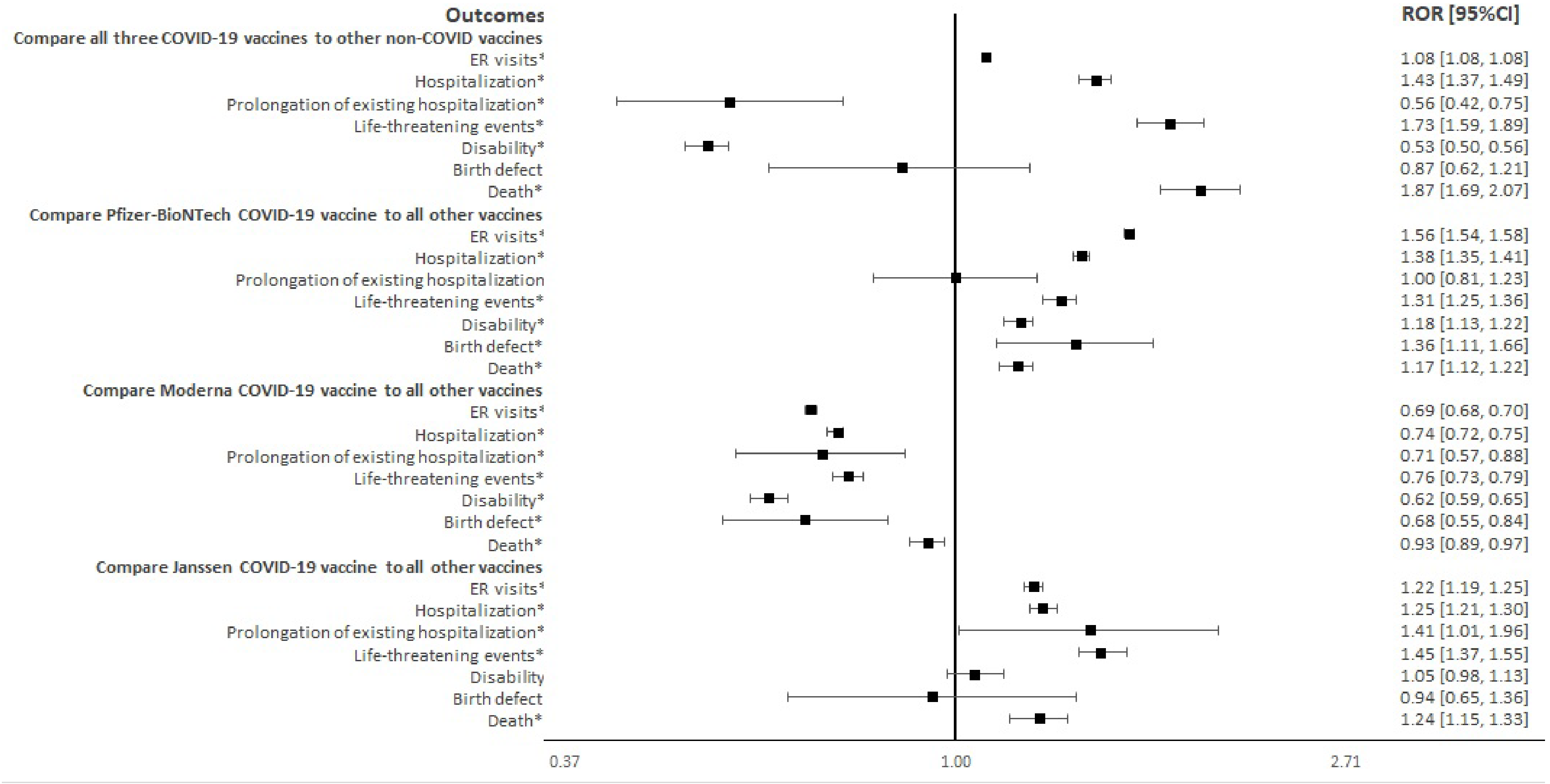

Supplement: Supplementary file 1 [file Data_Sheet_1.pdf]
